# The inhibitory effects of four inhibitors on the solution adsorption of CaCO_3_ on Fe_3_O_4_ and Fe_2_O_3_ surfaces

**DOI:** 10.1038/s41598-019-50127-x

**Published:** 2019-09-23

**Authors:** Changjun Li, Chaoyi Zhang, Wuping Zhang

**Affiliations:** 1 School of Petroleum and Natural Gas Engineering, Southwest Petroleum University, Chengdu, 610500 China; 2 CNPC Key Laboratory of Oil & Gas Storage and Transportation, Southwest Petroleum University, Chengdu, 610500 China; 3 Institute of Chemical Engineering, East China University of Science and Technology, Shanghai, 200237 China

**Keywords:** Reaction kinetics and dynamics, Energy

## Abstract

This study presents the inhibitory effects of four scale inhibitors, including polyacrylic acid (PAA), hydrolyzed polymaleic anhydride (HPMA), polyepoxysuccinic acid (PESA) and polyaspartic acid (PASP), on the adsorption of CaCO_3_ on the surfaces of Fe_3_O_4_ and Fe_2_O_3_. Samples were characterized using SEM and EDS and the average atomic number ratios of Ca/Fe were calculated. Inhibition effects followed the trend: PESA > PAA > PASP > HPMA and PESA > PASP > HPMA > PAA for Fe_3_O_4_ and Fe_2_O_3_, respectively. Molecular dynamics simulations based on the adsorption model of the scale inhibitor on the surface and calculations of the adsorption energy between the scale inhibitor molecule and the surface revealed that the relatively high scale inhibitory effect is due to low adsorption energy between the inhibitor molecule and the surface. Density Functional Theory (DFT) calculations of the model after adsorption revealed that the relatively low adsorption energy depends on the number of H-O bonds formed as well as those with higher Mulliken population values between the scale inhibitor and the surface.

## Introduction

Water produced from gas fields is a common byproduct in natural gas production. Typically, it is discharged into a post-treatment facility via sewage pipe after being separated from natural gas. Since the water produced contains a variety of ions, insoluble solid particles readily form via chemical reactions and adhere to the inner walls of the sewage pipe with CaCO_3_ serving as the prototypical example. As gas field sewage pipes are usually made of carbon steel, its surface readily oxidizes to Fe_3_O_4_ and Fe_2_O_3_ upon contact with sewage; Fe_3_O_4_ and Fe_2_O_3_ are the key oxidation products where scaling takes place.

The main treatment method for CaCO_3_ scale in gas fields is to add a chemical scale inhibitor (typically phosphate-free for environmental protection). Therefore, phosphorus-free scale inhibitors such as polyacrylic acid (PAA), hydrolyzed polymaleic anhydride (HPMA), polyepoxysuccinic acid (PESA) and polyaspartic acid (PASP) have been widely used. PAA can make the shape of CaCO_3_ in solution irregular^1–4^ and inhibits the preferential growth surface of CaCO_3_ crystals^2–4^. The effectiveness of CaCO_3_ inhibition is proportional to the concentration of PAA^1,3,4^. Meanwhile, the presence of PAA reduces the amount of CaCO_3_ precipitation on the rotating disk electrode by 70%^5^. HPMA inhibits the production of CaCO_3_ in solution, damages the regular shape of CaCO_3_^6^ and inhibits the preferential growth surface of CaCO_3_ crystals. Indeed, inhibition by HPMA is more effective than PAA^4^. PESA can also inhibit the formation of CaCO_3_ and damage the shape of CaCO_3_ in solution^7^. Molecular dynamics simulations suggest that PESA can adsorb on the preferential growth surface of CaCO_3_ crystals to inhibit their growth^8^. By comparing the scale inhibition efficiency, it was determined the inhibition efficiency of PESA on CaCO_3_ in solution was higher than that of HPMA and PAA^9^. PASP can also inhibit the formation of CaCO_3_ in solution and damage the shape of CaCO_3_^10,11^. However, the inhibition effect of PASP in solution is inferior to that of PESA^12^.

Previous studies have focused on the inhibition effect of scale inhibitors on CaCO_3_ in solution, while the effects of surface inhibition of CaCO_3_, especially on surfaces of Fe_3_O_4_ and Fe_2_O_3_, are not fully understood. In this study, we present an experimental simulation of surface CaCO_3_ scaling on Fe_3_O_4_ and Fe_2_O_3_ surfaces. The Ca/Fe ratios in different cases were obtained and compared with each other to evaluate the inhibition effects of the four inhibitors. We then established models of the scale inhibitor molecules with both the Fe_3_O_4_ and Fe_2_O_3_ surfaces using the Materials Studio.

The adsorption energies between the scale inhibitor and the surface were calculated and the results indicated that differences in the effects of the scale inhibitor in the scale inhibition process are attributed to the differences in the adsorption energy of the scale inhibitor molecules adsorbed on the surface. Finally, the number of chemical bonds and the Mulliken population values of inhibitor bonds with the Fe_3_O_4_ and Fe_2_O_3_ surfaces were calculated using DFT and the results indicated that the adsorption energy difference between the inhibitors and the surface are attributed to differences in quantity and Mulliken population value of chemical bonds.

## Experimental

### Materials

The No. 20 carbon steel used in this study (the same material as the sewage pipe of a gas field sewage station in Shandong, China) was cut into 50 × 25 × 2 mm^3^ cubes, immersed in ultra-pure water (UP water) at 51 °C (the station sewage temperature) for several days until the surfaces were completely/mostly black (Fe_3_O_4_) or orange (Fe_2_O_3_), and then dried.

The CaCl_2_ and NaHCO_3_ (AR, >96%) were purchased from Sichuan Kelong Company. Each group involved 0.933 g CaCl_2_ and 0.959 g NaHCO_3_ (yielding Ca^2+^ and HCO_3_^−^ concentrations of 0.336 g/L and 0.696 g/L, respectively). The concentrations of Ca^2+^ and HCO_3_^−^ were obtained from water quality testing of the sewage in the station pipe.

The concentrations of PAA, HPMA, PESA and PASP were 50% and purchased from Shandong Kerry Company. Each scale inhibitor was diluted to 1 g/L. Experimentally, 10 mL inhibitor solution was poured into the solution so that the concentration of the scale inhibitor in the test solution was 10 mg/L.

### Scaling

UP water was added to a beaker with an additional 30 mL UP water to compensate for evaporation loss (the evaporation loss amount was obtained experimentally). The water was heated to 51 °C on a stirring hotplate; CaCl_2_ and NaHCO_3_ were added to generate CaCO_3_.

**Figure 1:**
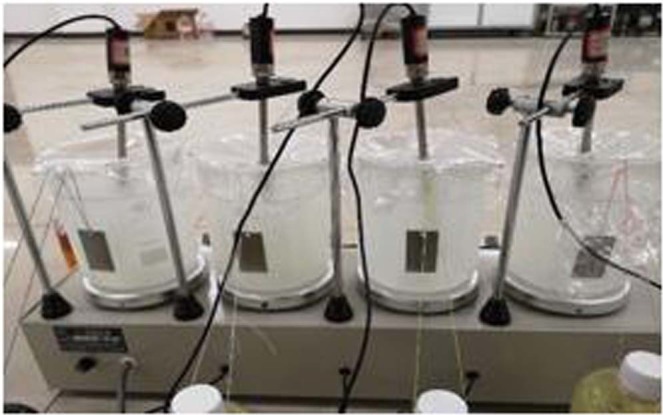
Experimental setup.

The quantities of CaCl_2_, NaHCO_3_ and scale inhibitor added in each group were:

1. CaCl_2_ + NaHCO_3_ + 1 L UP water;
2. CaCl_2_ + NaHCO_3_ + 0.99 L UP water + 10 mL PAA;
3. CaCl_2_ + NaHCO_3_ + 0.99 L UP water + 10 mL HPMA;
4. CaCl_2_ + NaHCO_3_ + 0.99 L UP water + 10 mL PESA;
5. CaCl_2_ + NaHCO_3_ + 0.99 L UP water + 10 mL PASP;

For Groups 2–5, the scale inhibitor was added 30 min after CaCl_2_ and NaHCO_3_ addition; a hanging piece of carbon steel (size 5 × 3) was added to the beaker 30 min after the addition of the scale inhibitor. The experiment lasted for 24 h. After 24 h, the hanging pieces were dried and purged. Trials involving hanging pieces of both Fe_3_O_4_ and Fe_2_O_3_ were repeated three times for each surface. The experimental set-up is shown in Fig. 1.

## Molecular Models and Simulation Details

### Software and force field

In this study, the Amorphous Cell, Discover, Forcite, and Caste modules in Materials Studio 7.0 software were used. The Amorphous Cell module was used to create a mixed layer of water molecules and scale inhibitor molecules. The Discover module was used to minimize energy while the Forcite module was used to run molecular dynamics simulation programs using the COMPASS force field^13–15^. The Castep module was used to calculate the bond number and the Mulliken population value between the scale inhibitor molecule and the surface, and its functionality is GGA and PBE^16,17^.

### Molecular models

**Figure 2:**
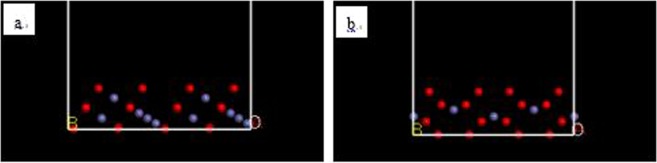
Models of the (111) surface (**a**) of Fe_3_O_4_ and (104) surface (**b**) of Fe_2_O_3_ (red ball: O atom, light blue ball: Fe atom).

In this study, the (111) surface^18–20^ of Fe_3_O_4_ crystals and the (104) surface of Fe_2_O_3_ crystals were examined as adsorption surfaces^21–23^. The initial molecular models of the Fe_3_O_4_ and Fe_2_O_3_ crystals were imported from a software database; the designated surface was cut to obtain the required adsorption surface. The a, b and c values of the (111) surface model of the established Fe_3_O_4_ crystal were 10.28 Å, 11.87 Å and 42.53 Å, respectively; the a, b and c values of the (104) surface model of the Fe_2_O_3_ crystal were 7.41 Å, 10.08 Å and 42.7 Å, respectively. All atoms on the surface were set in a fixed state. The surface model established is shown in Fig. 2.

**Figure 3:**
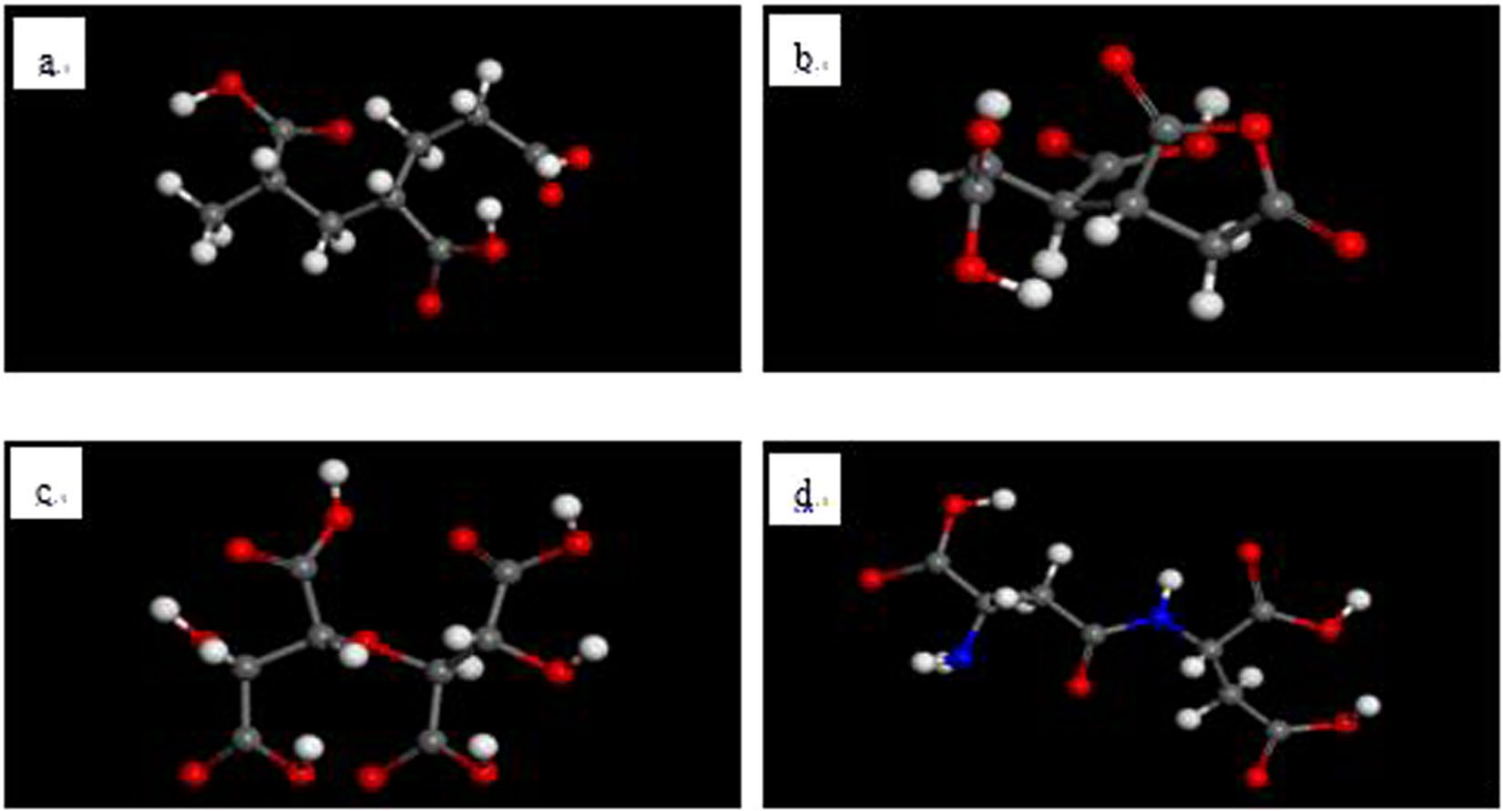
PAA (**a**), HPMA (**b**), PESA (**c**) and PASP (**d**) scale inhibitor model (red ball: O atom; white ball: H atom; gray ball: C atom; dark blue ball: N atom).

**Figure 4:**
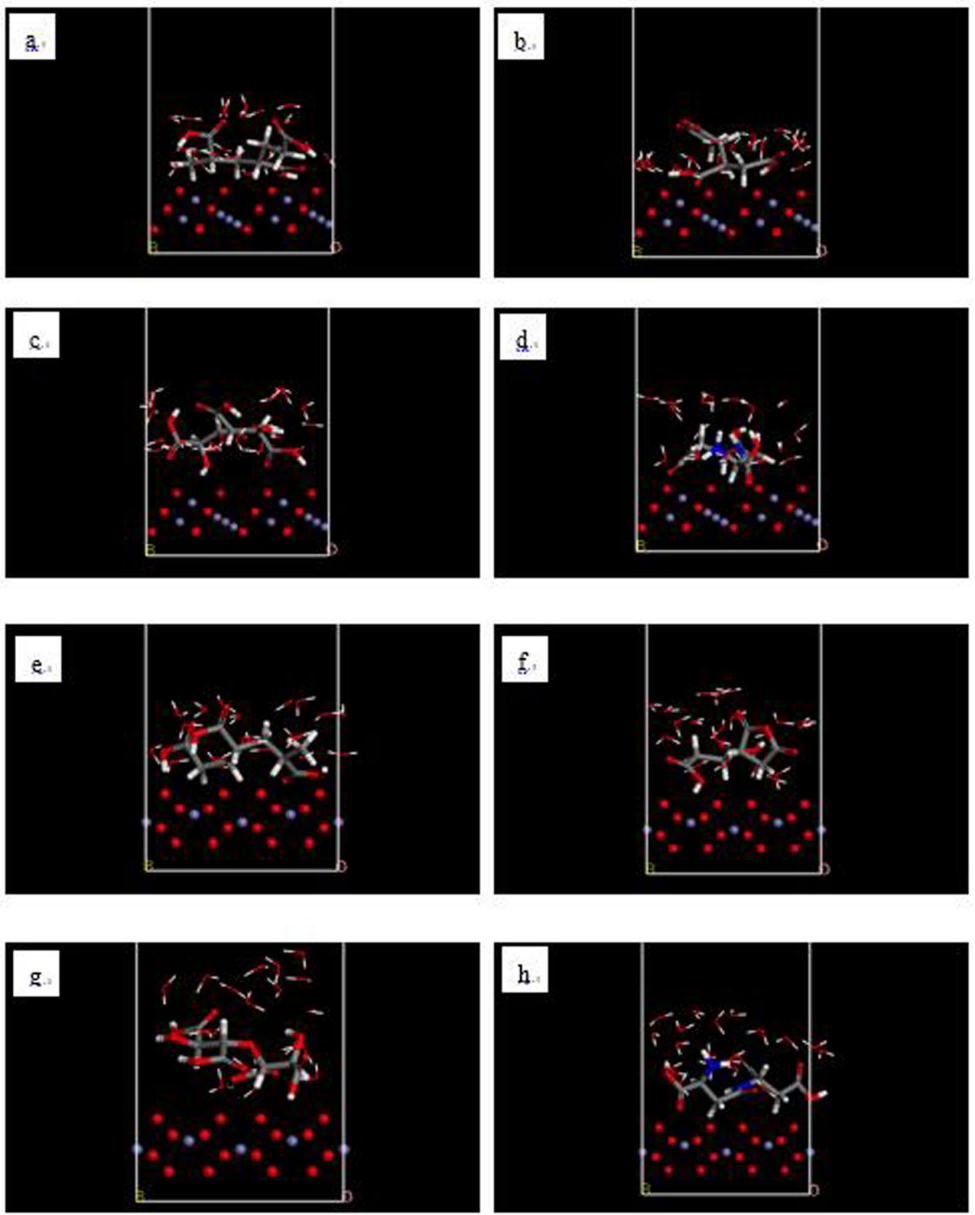
Initial models of adsorption of PAA molecules (**a**,**e**), HPMA molecules (**b**,**f**), PESA molecules (**c**,**g**) and PASP molecules (**d**,**h**) on the (111) surface of Fe_3_O_4_ crystals (**a**–**d**) and (104) surface of Fe_2_O_3_ crystals (**e**–**h**) (thick line: scale inhibitor; fine line: water molecules).

The four scale inhibitor molecules were manually drawn (see Fig. 3). Since adsorptions are in solution, a mixed layer was established in the Amorphous Cell module using a scale inhibitor molecule and 20 water molecules. The a and b values of the mixed layer are identical to the surface model values. The surface model was combined with the mixed layer by using the layer program and both the scale inhibitor molecule and water molecules were set in a free state^24^. The initial adsorption models of all inhibitors on both surfaces are shown in Fig. 4.

### Simulation

**Figure 5:**
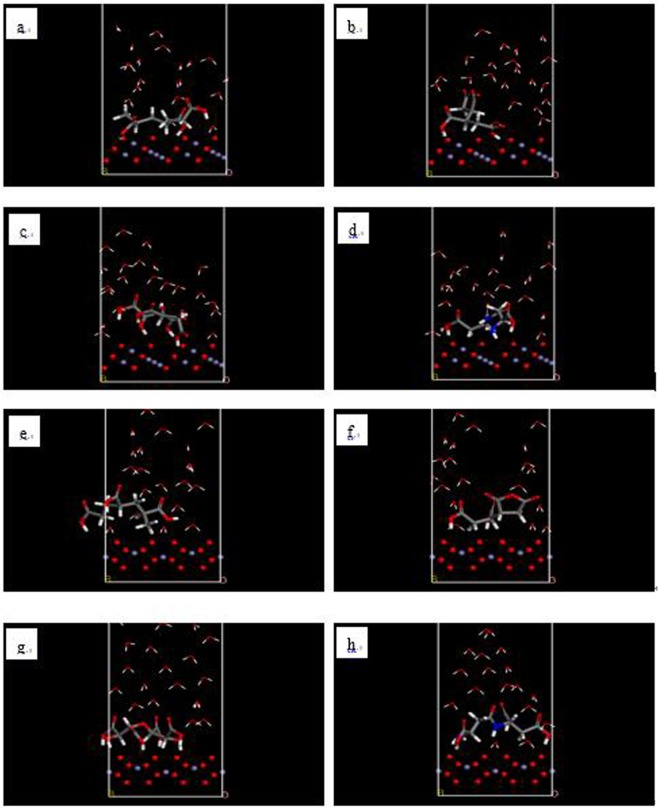
Final models of adsorption of PAA molecules (a,**e**), HPMA molecules (**b**,**f**), PESA molecules (**c**,**g**) and PASP molecules (**d**,**h**) on the (111) surface of Fe_3_O_4_ crystals (**a**–**d**) and (104) surface of Fe_2_O_3_ crystals (**e**–**h**) (thick line: scale inhibitor; fine line: water molecules).

After establishing the initial adsorption models, the energy was minimized using the discover module. Smart minimizer, which includes Steepest descent, Conjugate gradient and Newton, was selected as the energy minimization method in the module. The convergence of all methods was set at 10^−7^. The Forcite module was used for molecular dynamics simulation. The NVT ensemble was used, the temperature was 324 K (i.e., 51 °C), the number of steps calculated was 20,000,000 and Berendsen was selected as Themostat. The adsorption models of the scale inhibitor molecule on the (111) surface of Fe_3_O_4_ and the (104) surface of Fe_2_O_3_ from molecular dynamics calculations are shown in Fig. 5. Finally, the Castep module was used for DFT calculations. In this module, GGA and PBE were selected as Functional, and Fine was selected as Quality.

## Results and Discussion

### Surface characterization

A single hanging piece was set on a microscope carrier and two random points on the solution surface side of the 50 × 25 mm^2^ dimension were selected and simultaneously detected by SEM (Quanta 250, FEI Co., USA) and EDS (magnification 1500x). The SEM images of Fe_3_O_4_ and Fe_2_O_3_ steel hanging pieces are shown in Figs 6–17.

**Figure 6:**
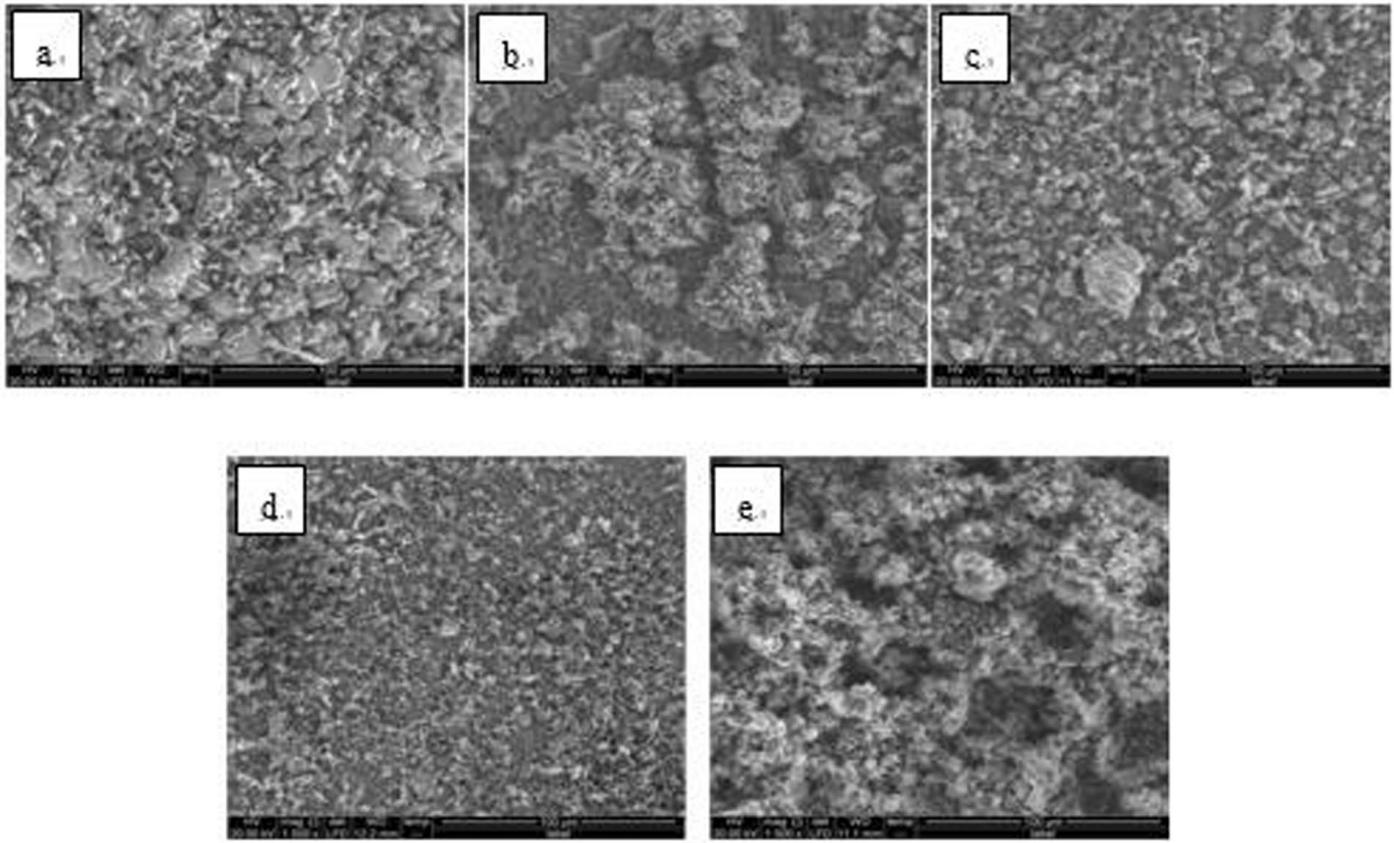
SEM images of detection point #1 of Fe_3_O_4_ hanging piece No. 1 (**a**: the solution does not contain scale inhibitor; **b**: the solution contains PAA; **c**: the solution contains HPMA; **d**: the solution contains PESA; **e**: the solution contains PASP).

**Figure 7:**
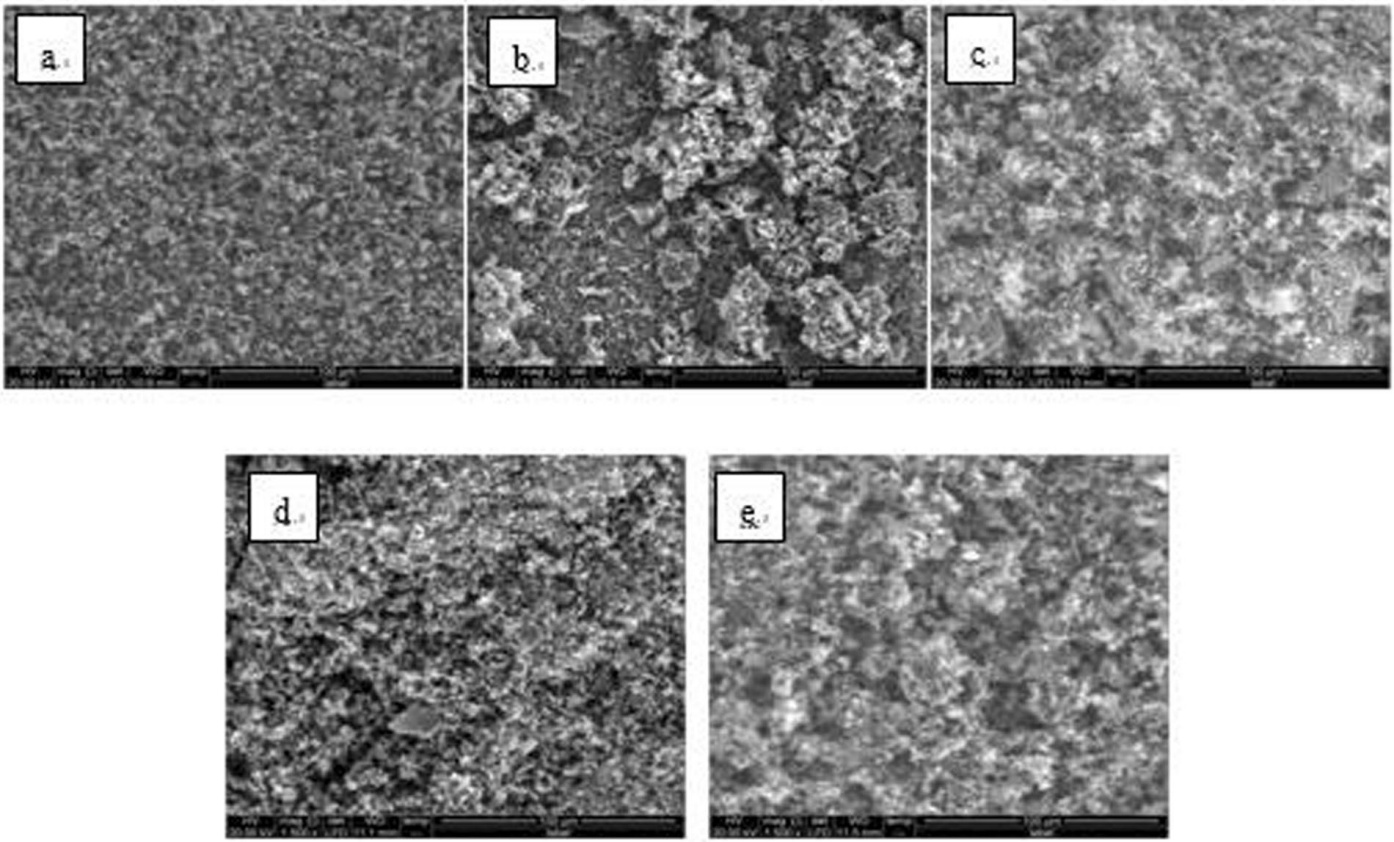
SEM images of detection point #2 of Fe_3_O_4_ hanging piece No. 1 (**a**: the solution does not contain scale inhibitor; **b**: the solution contains PAA; **c**: the solution contains HPMA; **d**: the solution contains PESA; **e**: the solution contains PASP).

**Figure 8:**
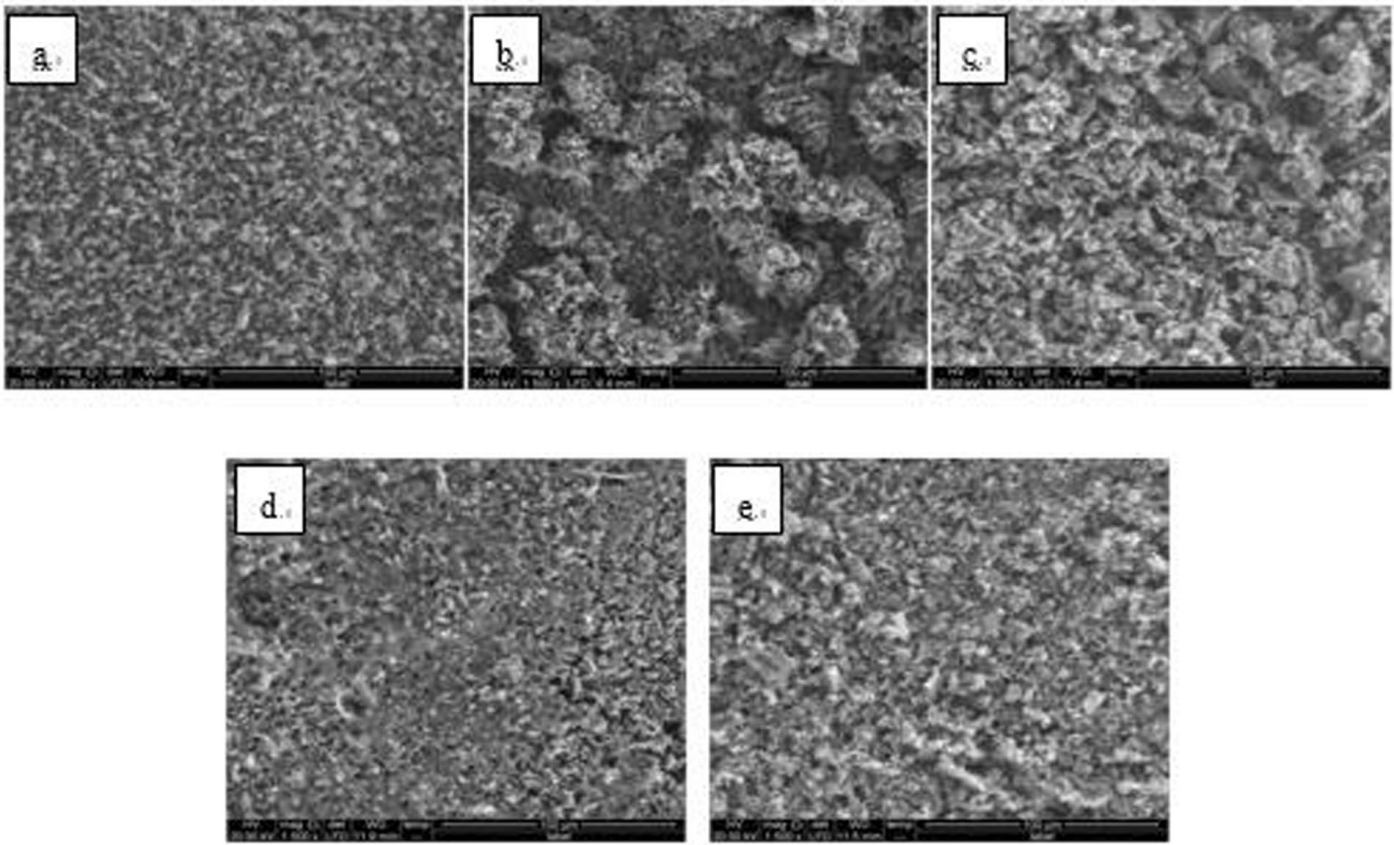
SEM images of detection point #1 of Fe_3_O_4_ hanging piece No. 2 (**a**: the solution does not contain scale inhibitor; **b**: the solution contains PAA; **c**: the solution contains HPMA; **d**: the solution contains PESA; **e**: the solution contains PASP).

**Figure 9:**
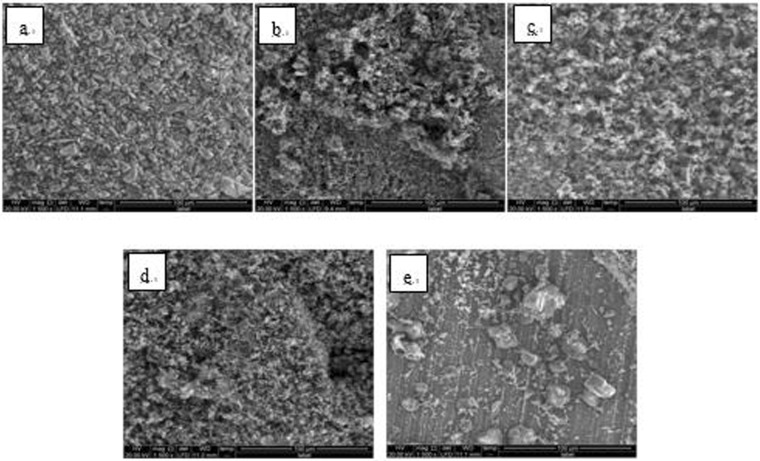
SEM images of detection point #2 of Fe_3_O_4_ hanging piece No. 2 (**a**: the solution does not contain scale inhibitor; **b**: the solution contains PAA; **c**: the solution contains HPMA; **d**: the solution contains PESA; **e**: the solution contains PASP).

**Figure 10:**
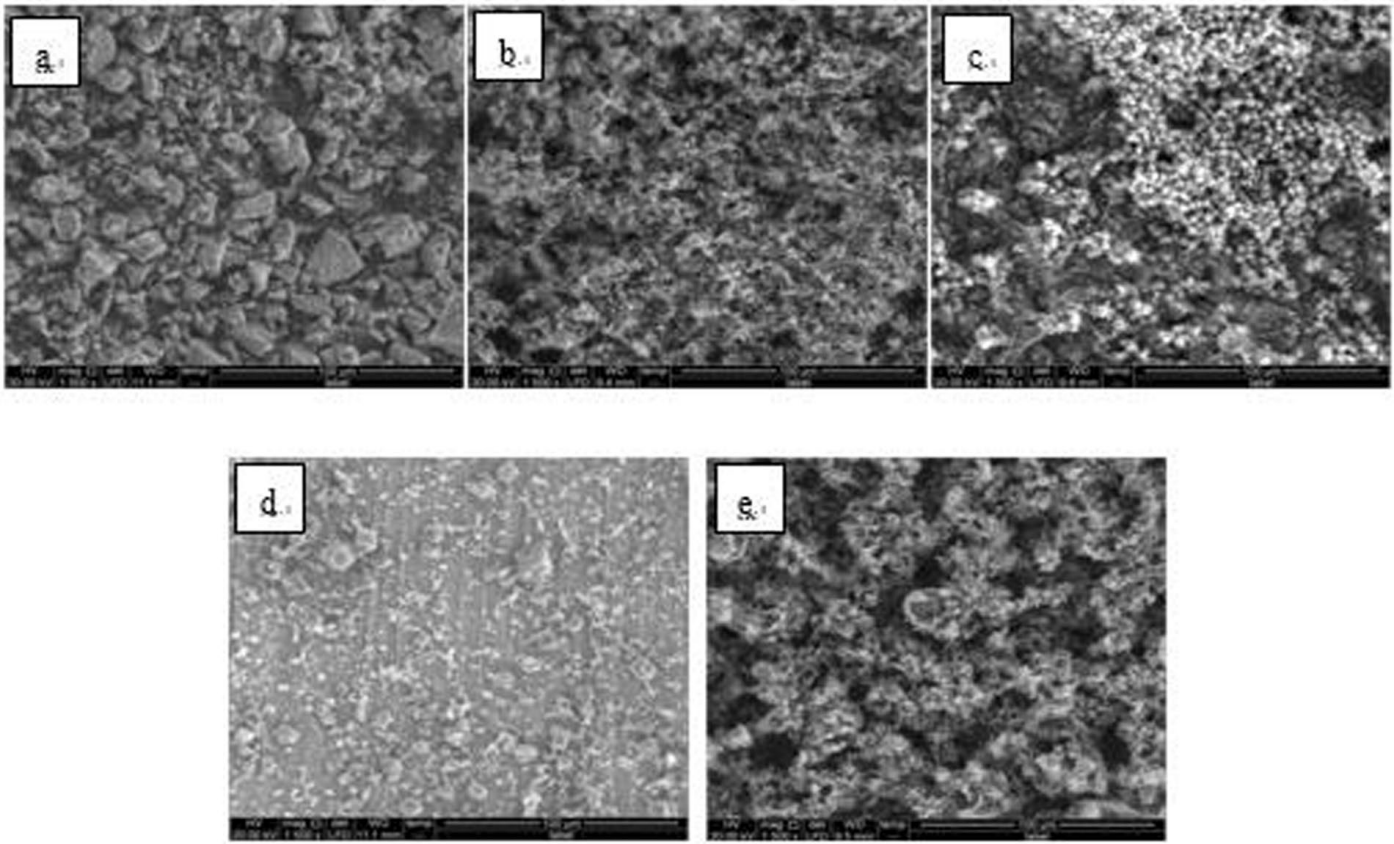
SEM images of detection point #1 of Fe_3_O_4_ hanging piece No. 3 (**a**: the solution does not contain scale inhibitor; **b**: the solution contains PAA; **c**: the solution contains HPMA; **d**: the solution contains PESA; **e**: the solution contains PASP).

**Figure 11:**
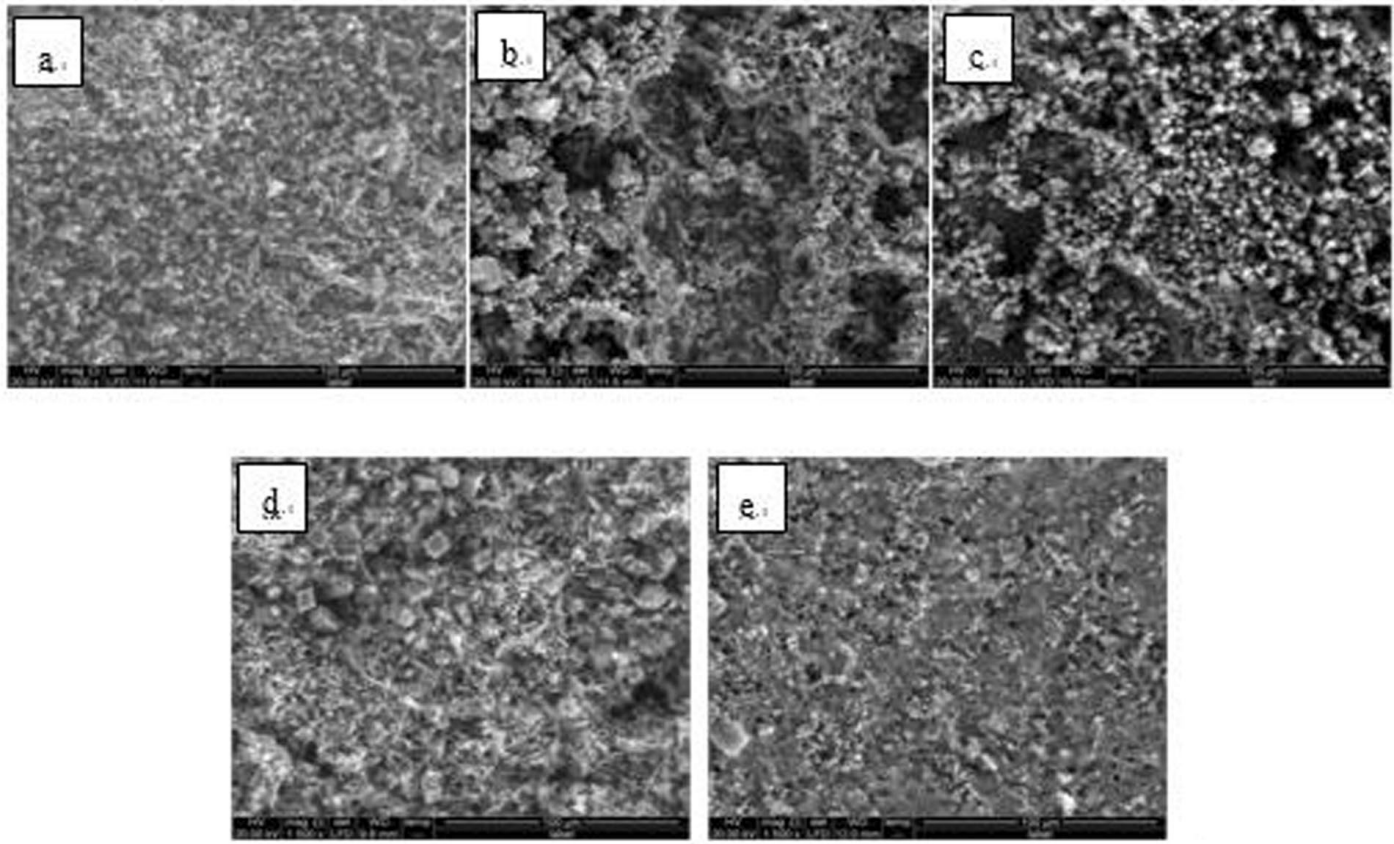
SEM images of detection point #2 of Fe_3_O_4_ hanging piece No. 3 (**a**: the solution does not contain scale inhibitor; **b**: the solution contains PAA; **c**: the solution contains HPMA; **d**: the solution contains PESA; **e**: the solution contains PASP).

**Figure 12:**
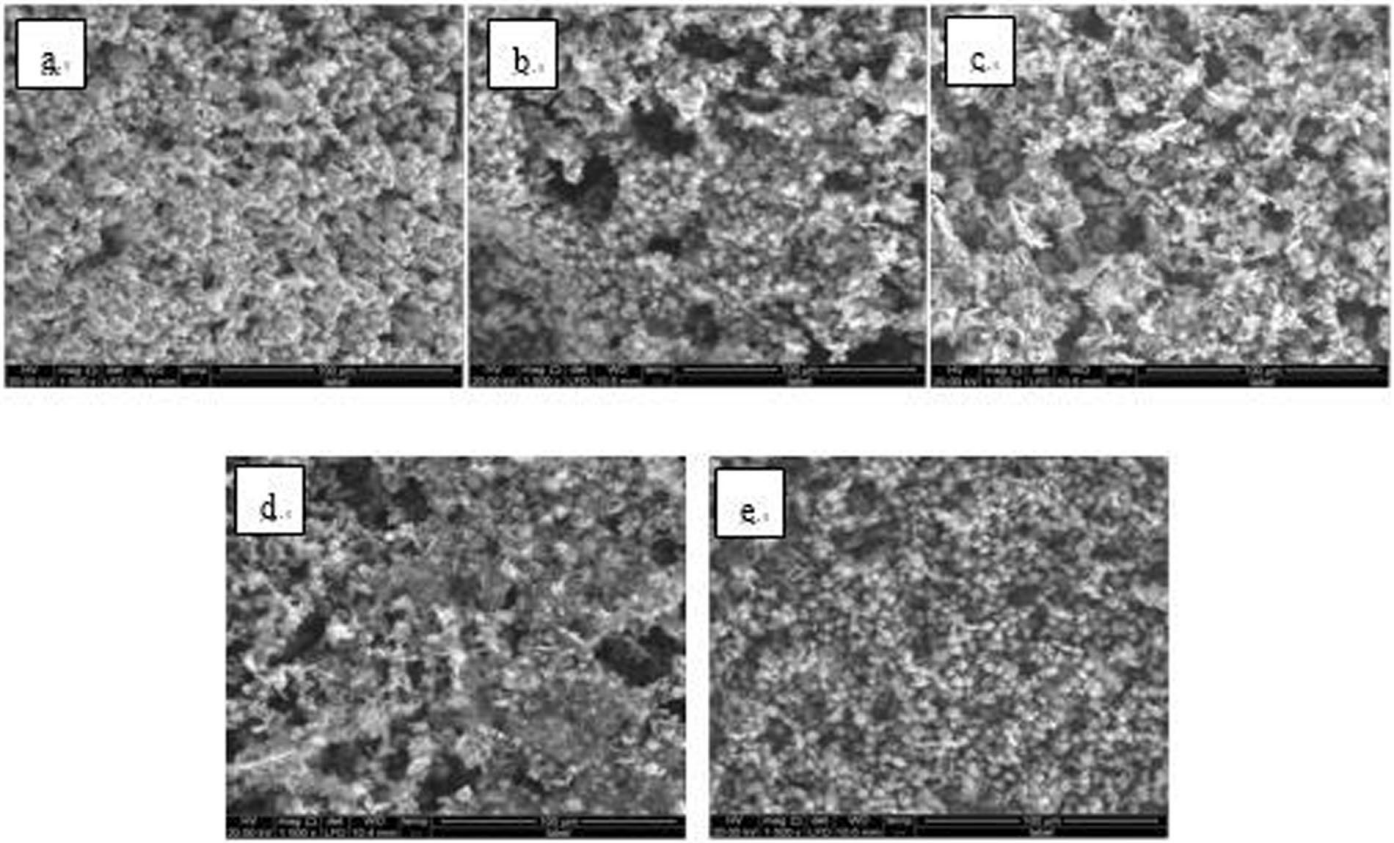
SEM images of detection point #1 of Fe_2_O_3_ hanging piece No. 1 (**a**: the solution does not contain scale inhibitor; **b**: the solution contains PAA; **c**: the solution contains HPMA; **d**: the solution contains PESA; **e**: the solution contains PASP).

**Figure 13:**
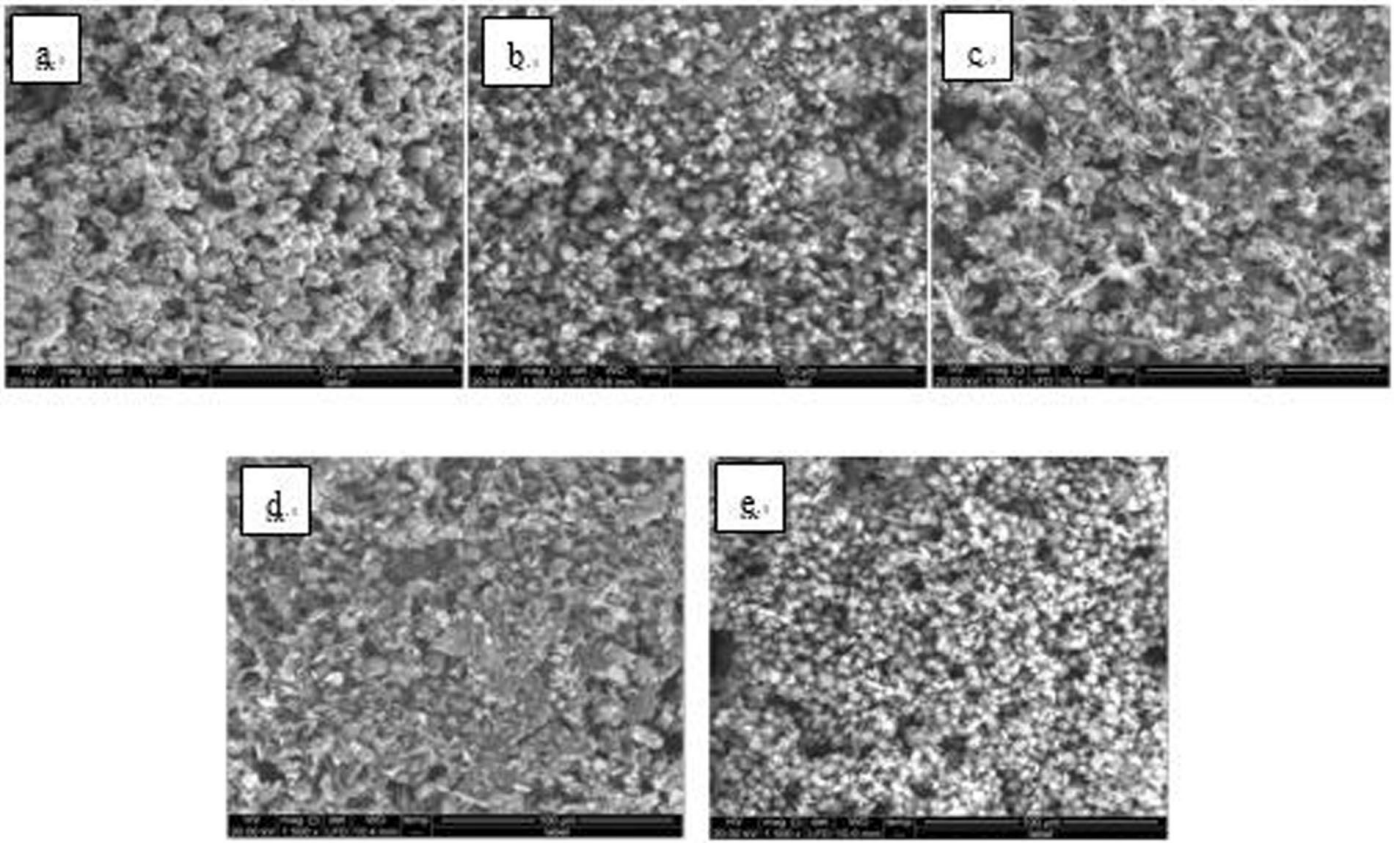
SEM images of detection point #2 of Fe_2_O_3_ hanging piece No. 1 (**a**: the solution does not contain scale inhibitor; **b**: the solution contains PAA; **c**: the solution contains HPMA; **d**: the solution contains PESA; **e**: the solution contains PASP).

**Figure 14:**
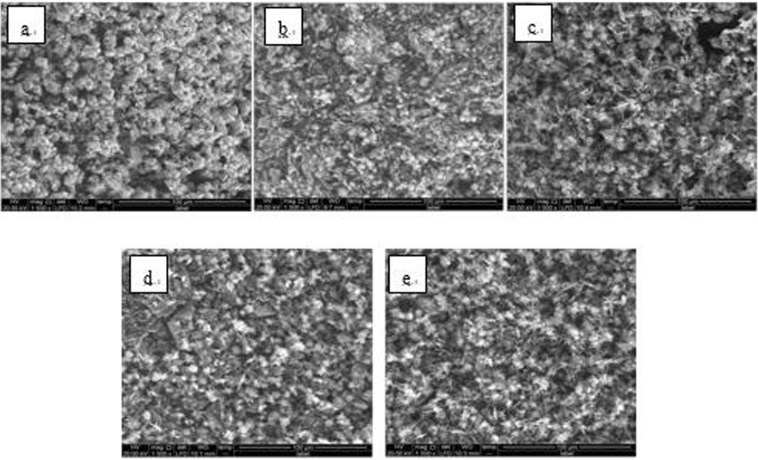
SEM images of detection point #1 of Fe_2_O_3_ hanging piece No. 2 (**a**: the solution does not contain scale inhibitor; **b**: the solution contains PAA; **c**: the solution contains HPMA; **d**: the solution contains PESA; **e**: the solution contains PASP).

**Figure 15:**
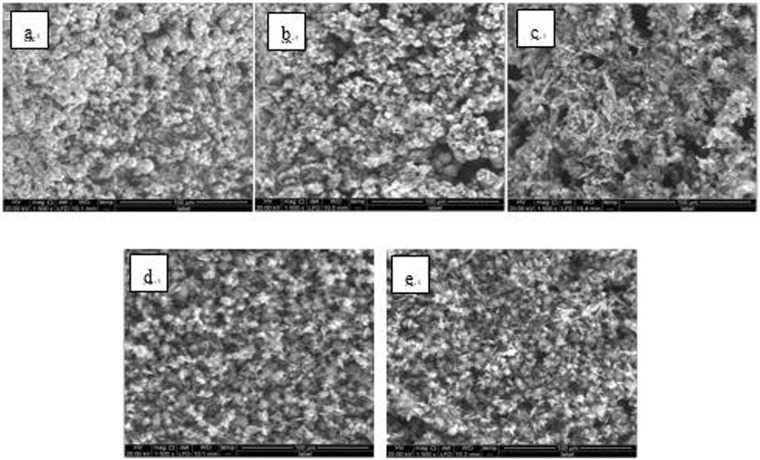
SEM images of detection point #2 of Fe_2_O_3_ hanging piece No. 2 (**a**: the solution does not contain scale inhibitor; **b**: the solution contains PAA; **c**: the solution contains HPMA; **d**: the solution contains PESA; **e**: the solution contains PASP).

**Figure 16:**
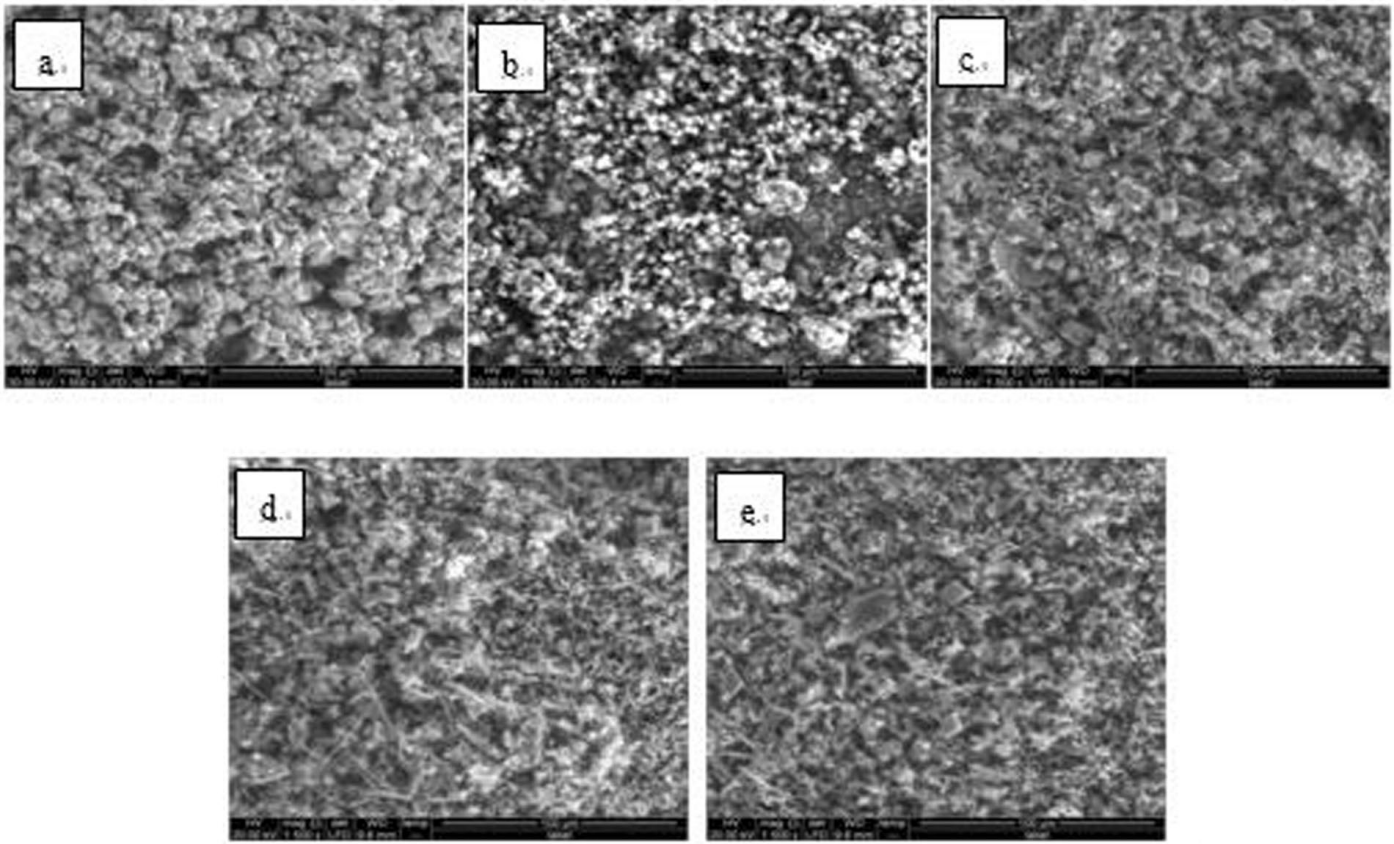
SEM images of detection point #2 of Fe_2_O_3_ hanging piece No. 3 (**a**: the solution does not contain scale inhibitor; **b**: the solution contains PAA; **c**: the solution contains HPMA; **d**: the solution contains PESA; **e**: the solution contains PASP).

**Figure 17:**
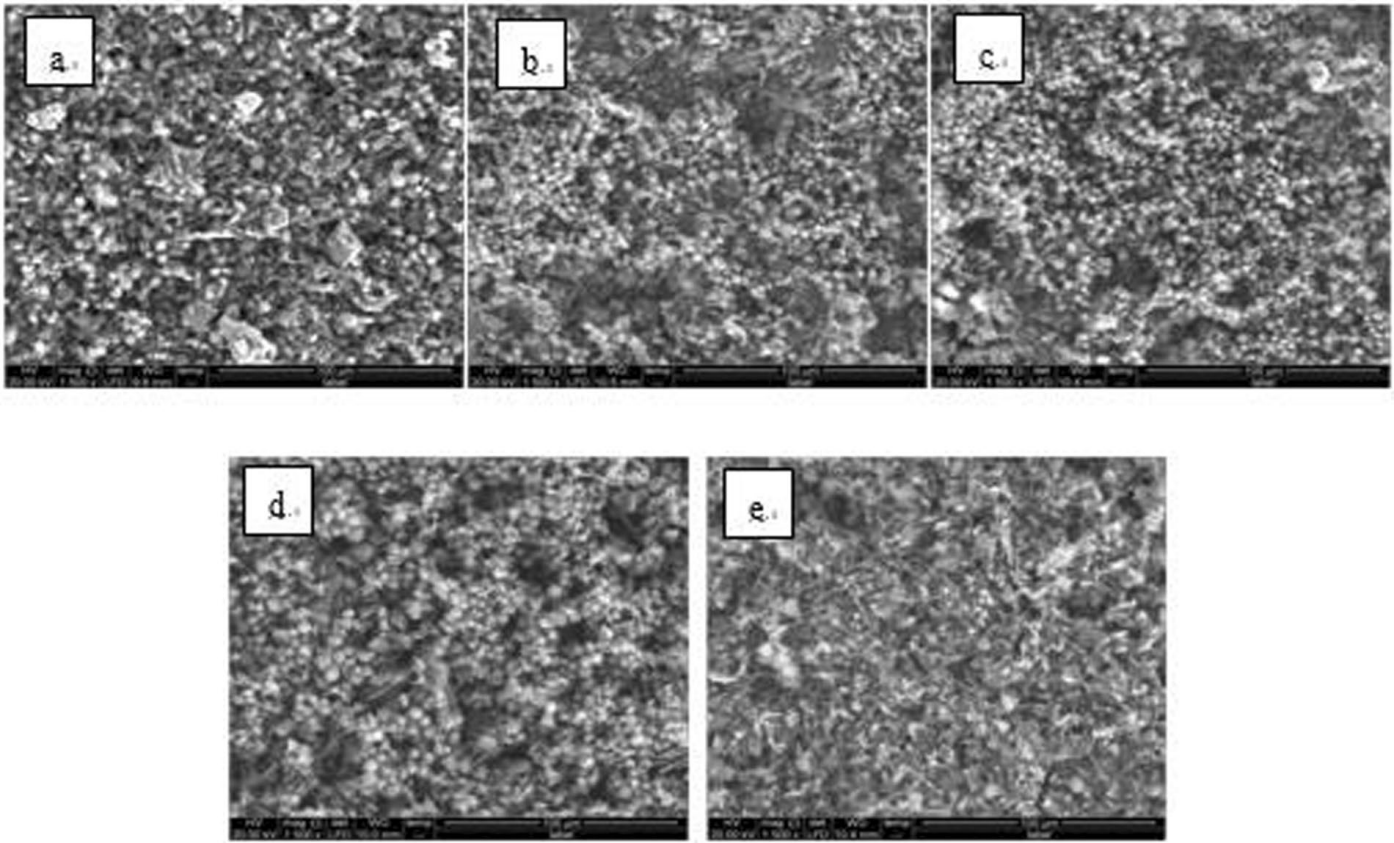
SEM images of detection point #2 of Fe_2_O_3_ hanging piece No. 3 (**a**: the solution does not contain scale inhibitor; **b**: the solution contains PAA; **c**: the solution contains HPMA; **d**: the solution contains PESA; **e**: the solution contains PASP).

As shown in Figs 6–17 CaCO_3_ covered almost the entire surface of the hanging piece in the absence of scale inhibitors, while a large number of “ditches” and “holes” were observed in the presence of scale inhibitors, indicating a reduced CaCO_3_ surface coverage.

### Energy dispersive spectroscopy (EDS)

The mass ratios and quantitative ratios of Ca and Fe on the detection points of the hanging pieces were obtained by EDS and are shown in Tables 1–4.

**Table 1:** EDS data of Ca and Fe adsorbed on the surface of Fe_3_O_4_ in a solution containing no scale inhibitor.

		Element	Atom Weight (%)	Atom Number (%)
hanging piece 1	detection point 1	Ca	37.21	19.13
		Fe	5.33	1.97
	detection point 2	Ca	40.95	23.61
		Fe	8.63	3.57
hanging piece 2	detection point 1	Ca	41.04	24.03
		Fe	10.05	4.22
	detection point 2	Ca	38.80	22.28
		Fe	10.05	4.14
hanging piece 3	detection point 1	Ca	36.96	21.4
		Fe	12.39	5.15
	detection point 2	Ca	30.55	19.04
		Fe	24.66	11.03

**Table 2:** EDS data of Ca and Fe adsorbed on the surface of Fe_3_O_4_ in a solution containing scale inhibitor.

Inhibitor			Element	Atom Weight (%)	Atom Number (%)
PAA	hanging piece 1	detection point 1	Ca	11.89	9.01
			Fe	56.31	30.63
		detection point 2	Ca	7.48	5.81
			Fe	61.87	34.51
	hanging piece 2	detection point 1	Ca	10.74	8.03
			Fe	56.34	30.25
		detection point 2	Ca	9.62	7.64
			Fe	61.53	35.03
	hanging piece 3	detection point 1	Ca	7.49	5.94
			Fe	63.28	36.01
		detection point 2	Ca	8.19	6.26
			Fe	60.14	33.03
HPMA	hanging piece 1	detection point 1	Ca	33.30	22.23
			Fe	28.30	13.56
		detection point 2	Ca	19.32	12.56
			Fe	37.87	17.68
	hanging piece 2	detection point 1	Ca	18.74	12.25
			Fe	38.81	18.21
		detection point 2	Ca	15.20	10.59
			Fe	47.03	23.51
	hanging piece 3	detection point 1	Ca	24.17	15.95
			Fe	35.04	16.6
		detection point 2	Ca	25.08	16.76
			Fe	35.3	16.93
PESA	hanging piece 1	detection point 1	Ca	1.54	1.51
			Fe	81.8	57.56
		detection point 2	Ca	3.32	2.88
			Fe	72.93	45.45
	hanging piece 2	detection point 1	Ca	4.83	4.04
			Fe	69.09	41.41
		detection point 2	Ca	4.84	3.87
			Fe	66.15	37.98
	hanging piece 3	detection point 1	Ca	9.96	8.69
			Fe	67.57	42.27
		detection point 2	Ca	13.21	9.75
			Fe	53.19	28.16
PASP	hanging piece 1	detection point 1	Ca	8.69	6.44
			Fe	57.29	30.45
		detection point 2	Ca	8.51	6.32
			Fe	57.66	30.73
	hanging piece 2	detection point 1	Ca	12.80	9.22
			Fe	51.70	26.72
		detection point 2	Ca	11.57	9.10
			Fe	59.26	33.44
	hanging piece 3	detection point 1	Ca	7.51	6.15
			Fe	65.43	38.41
		detection point 2	Ca	9.45	6.95
			Fe	56.08	29.58

**Table 3:** EDS data of Ca and Fe adsorbed on the surface of Fe_2_O_3_ in a solution containing no scale inhibitor.

		Element	Atom Weight (%)	Atom Number (%)
hanging piece 1	detection point 1	Ca	44.36	26.26
		Fe	8.31	3.53
	detection point 2	Ca	47.02	28.20
		Fe	7.26	3.12
hanging piece 2	detection point 1	Ca	46.37	27.57
		Fe	7.01	2.99
	detection point 2	Ca	46.39	27.78
		Fe	7.67	3.30
hanging piece 3	detection point 1	Ca	44.29	26.64
		Fe	9.85	4.25
	detection point 2	Ca	44.2	25.38
		Fe	5.5	2.26

**Table 4:** EDS data of Ca and Fe adsorbed on the surface of Fe_2_O_3_ in a solution containing scale inhibitor.

Inhibitor			Element	Atom Weight (%)	Atom Number (%)
PAA	hanging piece 1	detection point 1	Ca	34.90	21.84
			Fe	21.37	9.60
		detection point 2	Ca	11.10	8.01
			Fe	53.29	27.60
	hanging piece 2	detection point 1	Ca	26.84	17.31
			Fe	30.78	14.24
		detection point 2	Ca	20.64	14.12
			Fe	41.01	20.14
	hanging piece 3	detection point 1	Ca	21.2	14.26
			Fe	39.16	18.91
		detection point 2	Ca	33.55	21.83
			Fe	25.91	12.1
HPMA	hanging piece 1	detection point 1	Ca	25.02	16.25
			Fe	32.92	15.34
		detection point 2	Ca	32.08	20.04
			Fe	23.60	10.59
	hanging piece 2	detection point 1	Ca	18.11	12.84
			Fe	46.02	23.42
		detection point 2	Ca	8.82	6.99
			Fe	62.09	35.30
	hanging piece 3	detection point 1	Ca	25.08	16.37
			Fe	33.34	15.62
		detection point 2	Ca	24.02	15.5
			Fe	33.19	15.37
PESA	hanging piece 1	detection point 1	Ca	8.45	6.26
			Fe	57.47	30.54
		detection point 2	Ca	15.89	10.97
			Fe	45.71	22.64
	hanging piece 2	detection point 1	Ca	13.36	9.62
			Fe	51.16	26.43
		detection point 2	Ca	12.03	8.96
			Fe	54.92	29.37
	hanging piece 3	detection point 1	Ca	6.25	4.89
			Fe	63.35	35.56
		detection point 2	Ca	11.8	9.39
			Fe	59.89	34.19
PASP	hanging piece 1	detection point 1	Ca	18.80	13.06
			Fe	43.76	21.81
		detection point 2	Ca	14.36	10.69
			Fe	52.94	28.30
	hanging piece 2	detection point 1	Ca	11.39	8.38
			Fe	54.59	28.85
		detection point 2	Ca	13.00	9.64
			Fe	53.78	28.63
	hanging piece 3	detection point 1	Ca	7.03	5.44
			Fe	61.96	34.42
		detection point 2	Ca	20.25	13.6
			Fe	39.77	19.16

Tables 1–4 show that the ratios of CaCO3 areas and the surface area of the suspended pieces in different solutions were obtained based on the average ratio of Ca and Fe atoms at each detection point (Table 5).

**Table 5:** Average atomic number ratio of Ca/Fe in each solution.

Surface	Solution	Ca/Fe
Fe_3_O_4_	No inhibitor	5.547
	PAA	0.217
	HPMA	0.904
	PESA	0.14
	PASP	0.238
Fe_2_O_3_	No inhibitor	8.602
	PAA	1.173
	HPMA	0.993
	PESA	0.251
	PASP	0.423

Since the areas of all detection points are identical, the area occupied by CaCO_3_ increased and the scale inhibition effect degraded as the Ca/Fe ratio increased. As shown in Table 5, the Ca/Fe ratio in the absence of a scale inhibitor increased significantly relative to when an inhibitor was present.

In addition, the Ca/Fe ratios are different for different inhibitors. Indeed, the Ca/Fe ratios of the four inhibitors on the surface of Fe_3_O_4_ increase in the following manner PESA < PAA < PASP < HPMA, indicating that inhibition of CaCO_3_ scale on the Fe_3_O_4_ surface follows the same sequence. The Ca/Fe ratios of the four scale inhibitors on the surface of Fe_2_O_3_ follow the sequence of PESA < PASP < HPMA < PAA.

### Calculation of adsorption energy

The inhibition of CaCO_3_ surface adsorption by scale inhibitors is that active sites on the surface prefer occupation by the inhibitor molecules relative to CaCO_3_. The adsorption energy between the inhibitor molecules and the surface is calculated by^25,26^:

1 $$\Delta E={E}_{{\rm{surf}}+{\rm{inhi}}}-({E}_{{\rm{surf}}}+{E}_{{\rm{inhi}}})$$

where ${E}_{{\rm{surf}}+{\rm{inhi}}}$ refers to the model energy in the presence of both surfaces and scale inhibitor molecules; *E*_surf_ and *E*_inhi_ refer to the model energy in the presence of surface or scale inhibitor molecules, respectively. The adsorption energies between the four inhibitor molecules and the surfaces are shown in Table 6.

**Table 6:** Adsorption energies between the scale inhibitor molecule and the surfaces (kcal/mol).

Surface	Inhibitor	${{\boldsymbol{E}}}_{{\bf{surf}}{\boldsymbol{+}}{\bf{inhi}}}$	${{\boldsymbol{E}}}_{{\bf{surf}}}$	${{\boldsymbol{E}}}_{{\bf{inhi}}}$	${\boldsymbol{\triangle }}{\boldsymbol{E}}$
Fe_3_O_4_	PAA	−12854.455	−12593.864	−17.583	−243.008
	HPMA	−12807.105	−12593.864	−60.97	−152.271
	PESA	−12836.986	−12593.864	46.957	−290.079
	PASP	−12853.702	−12593.864	−18.21	−241.628
Fe_2_O_3_	PAA	−10708.272	−10463.753	−26.938	−217.581
	HPMA	−10751.637	−10463.753	−38.481	−249.403
	PESA	−10737.402	−10463.753	112.512	−386.261
	PASP	−10792.636	−10463.753	21.546	−350.429

All Δ*E* values in Table 6 are negative, indicating that adsorptions are spontaneous. As the adsorption energy decreased, the adsorption strength increased as did the adsorption stability. As shown in Table 6, Δ*E* follows the sequence of PESA < PAA < PASP < HPMA, indicating the adsorption strength of the inhibitors on the Fe_3_O_4_ surface increases PESA > PAA > PASP > HPMA. For Fe_2_O_3_, the Δ*E* increased in the following manner, PESA < PASP < HPMA < PAA which means the inhibitor adsorption strength on the Fe_2_O_3_ surface decreased in the following manner PESA > PASP > HPMA > PAA. As the adsorption strength increased, the stability of adsorption of the inhibitor on the surface increased. As a result, active sites on the surface are not easily occupied by CaCO_3_, enhancing scale inhibition. Therefore, the adsorption effects of the four inhibitors on the surfaces of Fe_3_O_4_ and Fe_2_O_3_ depend on the adsorption energy between the inhibitor and the surface.

As shown in Tables 5 and 6, the scale inhibition effect is related to the adsorption energy. The adsorption energies between Fe_3_O_4_ and inhibitors PASP and PAA were similar, as were the Ca/Fe ratios and inhibition effects. For the Fe_2_O_3_ surface, the PSAP and PESA adsorption energies were significantly lower than the adsorption energies of HPMA and PAA so the inhibitory effects and Ca/Fe ratios of PSAP and PESA were markedly lower for the Fe_2_O_3_ surface.

### DFT calculations

As the bonds between the inhibitor molecule and the surface increased and the bonding Mulliken population value increased, the binding affinity of the scale inhibitor molecule and the surface increased so the adsorption energy decreased. Therefore, the difference in adsorption energy between the inhibitor and the surface can be attributed to the number of bonds between the inhibitor molecule and the surface as well as the bonding Mulliken population value. The bonding between each inhibitor and the surfaces is shown in Table 7.

**Table 7:** Chemical bonds, bond Mulliken population values and bond length formed between the scale inhibitor molecule and the surfaces.

Surface	Inhibitor	Bond	Mulliken population	Length (Å)
Fe_3_O_4_	PAA	H16-O17	0.1	1.7657
		H9-O7	0.09	1.769
	HPMA	H9-O17	0.07	1.911
	PESA	H10-O2	0.14	1.5321
		H12-O7	0.12	1.6116
		H3-O14	0.11	1.6392
	PASP	H12-O2	0.11	1.5615
		H13-O14	0.06	1.9567
Fe_2_O_3_	PAA	H10-O7	0.13	1.5466
	HPMA	H9-O15	0.1	1.6576
		H8-O11	0.09	1.672
	PESA	H9-O3	0.15	1.5095
		H12-O7	0.13	1.5712
		H10-O15	0.13	1.5956
		H8-O11	0.12	1.6432
		H3-O5	0.05	1.8329
		H11-O12	0.04	1.8675
	PASP	H14-O7	0.14	1.5342
		H12-O11	0.14	1.5437
		H3-O11	0.03	1.8897

As shown in Table 7, H atoms are not present on the surface. Therefore, the bonds were formed by the H atoms in the adsorbent molecule and the O atoms on the surface.

Upon inhibitor adsorption on the Fe_3_O_4_ surface, three H-O bonds formed between PESA and Fe_3_O_4_ with Mulliken population values >0.1. Indeed, PESA was superior to the other three samples in terms of both the total number of H-O bonds and bonds with Mulliken population values >0.1. Hence, the adsorption strength of PESA on the Fe_3_O_4_ surface was the highest among all samples. PSAP and PAA each formed two H-O bonds with Fe_3_O_4_ and both had one bond with a Mulliken population value <0.1. Therefore, the adsorption of PAA and PASP on Fe_3_O_4_ surface was slightly weaker than PESA. The Mulliken population values of the two H-O bonds between PAA and Fe_3_O_4_ were slightly higher than the two H-O bonds between PASP and Fe_3_O_4_; therefore, the adsorption strength of PAA on Fe_3_O_4_ was slightly higher than PASP. However, only one H–O bond was generated between HPMA and Fe_3_O_4_ and its Mulliken population value was below 0.1. Hence, the adsorption strength of HPMA on Fe_3_O_4_ was the lowest among all inhibitor molecules. In summary, the adsorption strengths of scale inhibitors on Fe_3_O_4_ surface follow the sequence of PESA > PAA > PASP > HPMA, which is consistent with the sequence of adsorption energy.

Upon adsorption onto Fe_2_O_3_, 6 H-O bonds were generated between PESA and Fe_2_O_3_; four of them had Mulliken population values above 0.1. Indeed, PESA was superior to the other three samples in terms of both the total number of H-O bonds and bonds with Mulliken population values >0.1. Hence, the adsorption strength of PESA on Fe_2_O_3_ surface was the highest among all samples. PASP and Fe_2_O_3_ formed 3 H-O bonds and two of them had Mulliken population values above 0.1. Two H-O bonds were generated between HPMA and Fe_2_O_3_, and one of them had a Mulliken population value above 0.1. Therefore, the adsorption strength of PASP on Fe_2_O_3_ was lower than PESA, but higher than HPMA. As only one H-O bond was generated between PAA and Fe_2_O_3_, the adsorption strength of PAA on Fe_2_O_3_ was the lowest among all samples.

The adsorption energy between scale inhibitors and the surfaces clearly depends on the number of H-O bonds generated between the inhibitor, the surface and their Mulliken population values.

## Conclusions

This study presents a study of the inhibitory effects of PAA, HPMA, PESA and PASP on the adsorption of CaCO_3_ to the surfaces of Fe_3_O_4_ and Fe_2_O_3_. According to average Ca/Fe ratios obtained by EDS, the scale inhibition effect follows the sequence of PESA > PAA > PASP > HPMA and PESA > PASP > HPMA > PAA for Fe_3_O_4_ and Fe_2_O_3_ surfaces, respectively. The adsorption energies between the inhibitor molecules and the surface were calculated by molecular dynamics simulations. The sequence of adsorption energies is PESA < PAA < PASP < HPMA and PESA < PASP < HPMA < PAA for Fe_3_O_4_ and Fe_2_O_3_ surfaces, respectively. A low adsorption energy means strong inhibitor adsorption on the surface and inhibition depends on adsorption strength. Thus, these results demonstrated that excellent inhibition is due to low adsorption energy between the scale inhibitor and the surface. The number of bonds generated and their Mulliken population values calculated by DFT indicated that low adsorption energy depends on the formation of considerable H-O bonds with high Mulliken population values between the scale inhibitor and the surface.
